# Crosstalk during the Carbon–Nitrogen Cycle That Interlinks the Biosynthesis, Mobilization and Accumulation of Seed Storage Reserves

**DOI:** 10.3390/ijms222112032

**Published:** 2021-11-06

**Authors:** Manpreet Kaur, Yamini Tak, Surekha Bhatia, Bavita Asthir, José M. Lorenzo, Ryszard Amarowicz

**Affiliations:** 1 Department of Biochemistry, Punjab Agricultural University, Ludhiana 141027, Punjab, India; manibuttar12@gmail.com (M.K.); b.asthir@rediffmail.com (B.A.); 2 Agricultural Research Station, Agricultural University, Ummedganj, Kota 324001, Rajasthan, India; yaminitak1992@gmail.com; 3 Department of Processing and Food Engineering, Punjab Agricultural University, Ludhiana 141027, Punjab, India; surekhabhatia@gmail.com; 4 Centro Tecnológico de la Carne de Galicia, Parque Tecnológico de Galicia, 32900 San Cibrao das Viñas, Spain; jmlorenzo@ceteca.net; 5 Área de Tecnología de los Alimentos, Facultad de Ciencias de Ourense, Universidad de Vigo, 32004 Ourense, Spain; 6 Institute of Animal Reproduction and Food Research, Polish Academy of Sciences, 10-748 Olsztyn, Poland

**Keywords:** carbohydrates, germination, photoassimilates, source–sink, utilization

## Abstract

Carbohydrates are the major storage reserves in seeds, and they are produced and accumulated in specific tissues during the growth and development of a plant. The storage products are hydrolyzed into a mobile form, and they are then translocated to the developing tissue following seed germination, thereby ensuring new plant formation and seedling vigor. The utilization of seed reserves is an important characteristic of seed quality. This review focuses on the seed storage reserve composition, source–sink relations and partitioning of the major transported carbohydrate form, i.e., sucrose, into different reserves through sucrolytic processes, biosynthetic pathways, interchanging levels during mobilization and crosstalk based on vital biochemical pathways that interlink the carbon and nitrogen cycles. Seed storage reserves are important due to their nutritional value; therefore, novel approaches to augmenting the targeted storage reserve are also discussed.

## 1. Introduction

A plant seed contains an embryo, which is responsible for the emergence of seedlings or new plants, and it is not merely a structure for propagation and dispersal but the most frequently harvested plant tissue due to its accumulation of stored nutrients. Storage reserves are utilized by plants for growth and development throughout their life cycle. The photoassimilates resulting from the photosynthetic pathways synthesize the storage reserves in situ in multiple forms, mainly carbohydrates, proteins and lipids. Species differ in the type and amount of storage reserves accumulated in their seeds [1]. The precursors of storage reserves are translocated through the phloem from the mother plant to the developing seed, and sucrose is the main form of sugar transported to the developing tissue [2,3], which occurs through symplastic or apoplastic pathways to the maternal tissue of the seed and embryo, respectively. The sucrolytic pathway is the key step in the establishment of the prestorage accumulation stage, with sucrose hydrolyzed into end products [4] and preferentially partitioned into carbohydrates (mainly starch, which is the major carbohydrate reserve), proteins and lipids depending upon the type of seed. Cereals (wheat, rice and maize) possess a high content of carbohydrates [5], legumes (peas and beans) have a high content of proteins [6] and oil seeds (rape seed and castor) possess high lipid content [7]. During seed germination, the accumulated storage products are hydrolyzed into transported forms via gibberellic acid secreted by the embryo, which causes the hydrolysis of storage products by activating amylases, hydrolases, proteases and lipases through the activation of the MYB transcription factor by inhibiting DELLA proteins [8,9]. The hydrolysis of storage products mobilizes storage reserves, thereby signaling the start of the new life cycle through seedling establishment. The effectual mobilization and utilization of seed storage reserves and subsequent germination are indicators of seed quality. Storage reserve accumulation is an important factor in nutritional value, and these reserves represent the ultimate foundation of the human diet. Storage accumulation deposited mainly during seed germination is regulated under various physiological processes and unfavorable conditions.

This review focuses on the seed storage reserve composition, source–sink relation, transportation from maternal tissue to seed, partitioning of transported forms into different storage accumulations, hydrolysis of the stored forms following germination, reserve remobilization through metabolic processes to the developing tissue, utilization of seed reserves during seedling establishment and novel approaches to augmenting seed storage reserve accumulation.

## 2. Storage Reserves of Seeds

The carbohydrate composition of seeds consists of both simple and complex carbohydrates [10]. Starch is the major carbohydrate source of the human diet and represents the foremost polysaccharide accumulated in seeds in granules of different shapes and sizes that are not soluble in water [11]. In addition, other carbohydrates, such as cell wall polysaccharides in various plants [12], mixed-linkage glucans (e.g., *Brachypodium distachyon*) [13], mannans (e.g., *Cyamopsis tetragonoloba*) [14] and fructans (e.g., grasses and cereals), are also thought to act as seed storage polysaccharides. Raffinose and stachyose co-exist in some seeds as free sugars. In mature soybean seeds, sucrose, stachyose and raffinose constitute approximately 8%, 5% and 2% of the seed dry weight, respectively, and may contribute to the survival of the seed in the dry state [15,16]. Galactomannans are typically restricted to the endosperm of legumes, but they may also occur in many non-leguminous families, such as Compositae and Convolvulaceae [17,18,19,20]. The ratio of mannose to galactose, however, varies from species to species. In seeds of fenugreek and guar, the endosperm is nonliving and the cells are almost totally filled with galactomannans [21], and in the seeds of *Ceratonia siliqua*, galactomannans are observed as thickenings in the walls of living endosperm [22]. Among mannans, glucomannans are of particular importance, and many reviews have covered the properties, applications and health benefits of various glucomannans from different sources [23,24,25,26]. Xyloglucans are found in the primary cell walls and the cotyledons of some dicotyledonous seeds, where they function as storage polysaccharides. Galactose-containing molecules were first detected in the cell walls of seeds in 1892 [27], and galactans were identified as a discrete polysaccharide in white lupin seeds in 1947 [28].

The presence of proteins in seeds varies depending on the type of seed in question, with 70% present in legumes [29] and up to 50% present in cereals [30]. Plant-based proteins form a major source of dietary protein, and seed storage proteins are of great significance due to their high nutritional quality and numerous functional properties [31]. For instance, the gluten storage protein in wheat is the primary reason why wheat flour is used in the baking process of bread and pasta [32]. The pseudocereal quinoa flour, however, is preferred in the flour industry for gluten-free products [33,34,35]. Moreover, the superior freeze–thaw capability of quinoa makes it useful in frozen foods [36]. Quinoa has better expansion properties and water holding capacity than wheat and barley, and has more viscous starch, making it useful as a thickening agent and in baby foods [34,37]. Seed storage proteins are broadly classified into albumins, globulins and prolamins. Albumins and globulins are important because of their high nutritional balance, presence of essential amino acids and high content of lysine [38], whereas prolamins are present in the endosperm and possess low nutritional value because they lack essential amino acids [39]. The pseudocereal quinoa has been reported to be a complete protein as it comprises all nine of the essential amino acids [40,41,42]. Albumins are usually dispersed in seeds of dicot plants and have been broadly identified in *Cruciferae*, particularly oilseed rape and *Arabidopsis*. Globulins are the most widely distributed group of storage proteins, and they are found in dicots, monocots and fern spores. They have also been examined in legumes, especially peas, French beans, broad beans and soybeans [43]. Based on their sedimentation coefficients, globulins can be classified into two groups: 11S and 7S globulins. Prolamins are mainly restricted to grasses, while albumins and globulins are widely distributed in flowering plants. In cereals, typically half of the total nitrogen content of grain has been formed by prolamins [30], which are is considered the largest storage protein type in wheat, corn and sorghum; moreover, their extraction from byproducts has been a popular research topic [44]. In oats and rice, however, there are lower amounts of prolamins present, comparatively speaking [45]. Depending on the sequence of amino acids, prolamins are classified into different groups, such as S-rich, S-poor and high molecular weight (HMW) prolamins. Based on the different cereal types, prolamins can be identified by different names, e.g., glutenins and gliadins (in wheat) [46], zeins (in corn) [47], kafirins (in sorghum) [48] and hordeins (in barley) [49].

Lipids are generally stored as triacylglycerols and represent an essential carbon storage form in many angiosperm seeds. Up to 80% of the total dry matter in some storage organs, such as nuts, cashews and hazelnuts, is composed of lipids [50,51]. They are water insoluble and deposited in discrete oil bodies, predominantly within seed storage tissue and in certain fruits, such as avocados and olives [52]. Palmitic and stearic acids are the chief fatty acids among various commercial vegetable oils [53]. Oleic and linoleic acids can also be abundant in some commercial oils [54], but linolenic acid is apt to oxidize quickly and produces off-flavours; hence, it is not suitable for cooking oil [55]. Seed oils have a high potential value due to their astonishing natural diversity and are widely used for nutritional or industrial purposes [56,57]; for instance, medium-chain-length fatty acids in coconuts and palm kernels show detergent-like properties [58], and long-chain erucic acid in rapeseed and ricinoleic acid in castor beans are valuable lubricants [59,60]. The liquid wax alcohol esters and long-chain fatty acids of jojoba seeds can be used as a substitute for whale oils and are used as lubricants in jet engines and enhancers in perfumes [61].

## 3. Seed Formation, Development and Filling

In higher plants, seed development is a crucial process that links two different sporophytic generations, and this genetically programmed process converts carbon and nitrogen precursors into stable reserves that go on to play a vital role in various processes [62]. Repeated cell division after double fertilization in the embryo sac leads to the establishment of triploid endosperm and diploid zygotes. Mature ovules develop into seeds surrounded by maternally derived seed coats (ovule integuments) and consist of embryos, endosperm or cotyledons. The development of seeds encompasses a coordinated patterned program that includes formation, deposition of storage reserves and desiccation [63]. During seed filling, the water content drops, the storage reserves accumulate and the cell expands. Monomers of various macromolecules are then imported through the phloem sieve tube elements and various biochemical processes occur during the synthesis of their storage form. The weight, size and nutritional quality of the seed are affected by the seed filling rate and duration.

## 4. Source and Sink Relation

Photosynthesis is an amphibolic process that occurs in the green parts of plants, and it plays an essential role in the formation of the storage reserves of seeds [64] and photosynthates. Sugars are the end products produced during the process. Thus, the sites of photosynthesis in plants where photoassimilates are produced represent the translocation source tissue, with photoassimilates traveling to the so-called sink tissues, wherein they are metabolized by various catabolic, amphibolic or anabolic reactions and then stored as seed reserves. Phloem sieve tubes of vascular bundles are responsible for translocation, and the partitioning of photosynthates, primarily sucrose, occurs during unloading in the sink tissues by specific processes [65]. Maternal symplasm is the major pathway that directs nutrients to the seed [66]. However, depending on the type of species and structure of the seed, there are various pathways for nutrient flow; for instance, sucrose is imported via the apoplastic tissue surrounding the embryo [67,68]. In sink tissue, sucrose is transformed into hexoses, such as glucose, fructose and UDP-glucose, based on specific enzymes, and these monosaccharides are vital for the development of seeds. It has been demonstrated in Arabidopsis that mutants of glucose affect seed development because they lead to the production of seeds with altered compositions of seed storage nutrients [69].

## 5. Sucrolytic Route Leading to Seed Storage Reserve Biosynthesis

**Figure 1:**
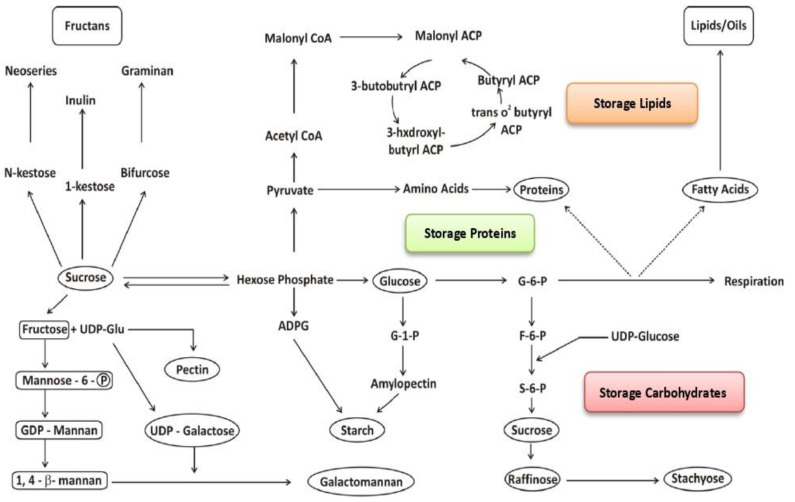
Crosstalk of various biochemical pathways during seed storage reserve biosynthesis starting from the precursor sucrose. Abbreviations: ADPG: ADP-glucose; F-6-P: fructose-6-phosphate; G-1-P: glucose-1-phosphate; G-6-P: glucose-6-phosphate; S-6-P: sucrose-6-phosphate; UDP-Glu: UDP-glucose.

In the developing seed, sugars (predominantly sucrose) that arrive are processed for storage reserves through an active and complex metabolism. During seed maturation, cleavage of sucrose elicits a metabolic switch, and the prestorage stage is established by the production of hexoses through the sucrolytic pathway with the help of hydrolyzing enzymes [4]. The process of translocation and metabolism of sugars is partitioned in seeds, and slight alterations in the ratio of hexose/sucrose can dramatically affect the pathways of storage metabolism and seed development [70]. Sucrose is hydrolyzed into fructose and glucose or UDP-glucose and fructose by invertase or sucrose synthase enzymes, respectively, and the formed fructose may be converted into glucose by isomerase enzymes or formed into hexose phosphate, which ultimately converts to glucose. Through an energy-dependent process, glucose molecules accumulate to form starch. Sugars are mainly stored in the form of starch [71] because greater energy can be packed into a smaller space in starch molecules compared with sucrose or glucose molecules; moreover, starch molecules can release this energy easily, thereby maximizing both mobilization and storage. Nucleotide sugars, e.g., UDP-glucose, can combine with fructose-6-phosphate and be stored in the form of galactomannans and mannans through a series of biochemical reactions. Liepman [72] has demonstrated that cellulose synthase-like enzymes can catalyze the biosynthesis of both mannans and glucomannans. The biosynthetic pathways for the synthesis of seed storage carbohydrates are elucidated in Figure 1. Sucrose also acts as the precursor for the biosynthesis of fructans, and the process is mediated by several fructosyltransferases [10], e.g., sucrose:sucrose-1-fructosyltransferase, fructan:fructan-1-fructosyltransferase, sucrose:fructan-6-fructosyltransferase and fructan:fructan-6G-fructosyltransferase, thus leading to the formation of different types of fructans depending on the seed type.

### 5.1. Protein Biosynthesis

Protein biosynthesis from imported sugars begins with glucose, which is obtained from the breakdown of sucrose. This biosynthesis occurs through a complex variety of intermediary metabolic pathways in the cytoplasm and lumen of the ER. Higher plants generally accumulate long-term reserves of nitrogen during maturation in the form of storage proteins, which are primarily localized in protein bodies (for zien and prolamin storage) and protein storage vacuoles (for globulin and albumin storage). Seed storage protein precursors are synthesized on the rough endoplasmic reticulum and then targeted to different locations [73]. Seed storage proteins are usually secretory proteins that possess a signal sequence at the N-terminus, which helps target the protein to the appropriate location, where it exerts its function or accumulates [74]. After translocation to the lumen of the ER, the signal sequence is cleaved, and newly synthesized proteins are directed, via the Golgi apparatus and trans-Golgi network, from the site of translation, i.e., the ER, to the storage sites, where they exert lytic activity or are degraded.

### 5.2. Lipid Biosynthesis

Lipid biosynthesis is a multifaceted and multistep process that occurs in three different cell compartments. The phosphorylation of glucose, fructose and free hexoses by glucokinases, fructokinases and hexokinases, respectively, directs the flow of carbon to the synthesis of fatty acids and the accumulation of oils [4]. The sucrose translocated from the photosynthetic tissue to the storage tissue of the seed acts as the precursor for the synthesis of lipids. In the seed storage tissue, glucose 6-phosphate or malate formed from the transported sucrose is used by the chloroplast, and glycerol 3-phosphate is used in the assembly of triacylglycerol. In certain oil seeds, plastids take up phosphoenolpyruvate, which serves as the substrate for the synthesis of lipids and subsequently forms acetyl-CoA [75]. The first committed step of fatty acid biosynthesis is the carboxylation of acetyl-CoA with the help of acetyl-CoA carboxylase to yield malonyl-CoA [76], which is then transferred to an acyl carrier protein. Following malonyl-acyl carrier, proteins are assimilated into the complex, and by consecutive addition the size of fatty acids increases by two carbons. Palmitic and stearic acids are usually the final products of the fatty acid synthase complexes [77], while shorter-chain fatty acids can also be formed in some seeds with the help of thioesterases, which terminate the reactions earlier. CoA-linked palmitic, stearic and oleic acids are translocated to the ER lumen, where additional fatty acid modifications as well as acyl chain elongation and many desaturase reactions occur, thus leading to the synthesis of nonconjugated, conjugated and hydroxylated polyunsaturated fatty acids. The interlinking of lipid biosynthesis from carbohydrate precursors is demonstrated in Figure 1.

## 6. Mobilization of Storage Reserves Following Germination

**Figure 2:**
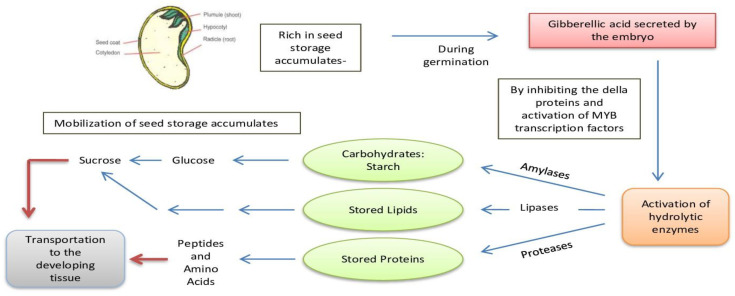
Generalized seed reserve mobilization in germinating seedlings.

During seed germination, gibberellic acid secreted by the embryo activates the hydrolytic enzymes present in the aleurone layer, i.e., amylases, hydrolases, proteases and lipases [9], by inhibiting DELLA proteins and activating MYB transcription factors [8]. Hydrolytic enzyme activation promotes the breakdown of storage reserves, including cell wall storage polysaccharides, following germination; for instance, it has been confirmed that legume mobilization of galactomannans is also accomplished through hydrolysis [78]. Simultaneously, amylase activation produces sucrose, which disassembles polysaccharides into their constituents. Ultimately, sucrose is the transport sugar involved in the mobilization (carbon and energy) of storage reserves to the growing embryo. The generalized seed storage mobilization process is depicted in Figure 2.

### Stored Lipids

**Figure 3:**
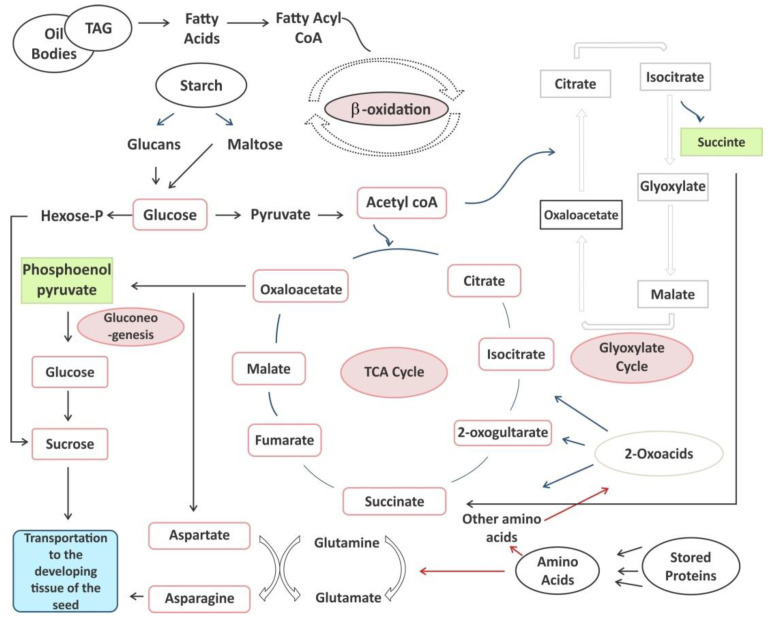
Interlinking of the carbon (sugars and lipids) and nitrogen (amino acids) cycles during seed germination.

Stored lipids serve as respiratory substrates and are transformed into sugars [79,80]. Lipolysis, β-oxidation, the glyoxylate cycle, the TCA cycle and gluconeogenesis are biochemical pathway sequences that occur during this process [81]. Seed-stored lipid mobilization begins with the breakdown of oil body-accumulated triacylglycerols into free fatty acids and monoacylglycerols with the help of lipases [82,83]. Subsequently, the β-oxidation of fatty acids occurs in mitochondria or peroxisomes [84], followed by the glyoxylate cycle, which partially occurs in the peroxisome and partially occurs in the cytoplasm. Fatty acids undergo ATP-dependent acylation reactions to form fatty acyl CoA in the cytosol, which enters mitochondria through the carnitine shuttle for β-oxidation and yields acetyl-CoA. Acetyl-CoA enters the glyoxylate cycle and tricarboxylic cycle in glyoxysomes and mitochondria, respectively. Succinate is synthesized in glyoxysomes and transported into mitochondria, which is followed by conversion to malate through the TCA cycle (Figure 3). In turn, oxaloacetate is formed from succinate transported in the cytosol. The final step of stored lipid breakdown is the synthesis of sugars, through which carbon transport occurs through gluconeogenesis. However, the interplay between carbon and nitrogen metabolism is significant in seeds, which can accumulate bulky amounts of stored proteins as well as lipids. The interlinking of the carbon (sugars and lipids) and the nitrogen cycle is described in Figure 3. Before stored lipid breakdown, carbon atoms are incorporated into asparagine, aspartate, glutamine and glutamate, which are specifically involved in the biosynthetic pathways of primary amino acids, whereas during germination of legume seeds, large amounts of ammonia are produced from the mobilization of storage proteins [85]. In lipids, ammonia is temporarily stored in asparagine, and it may account for 30% of the dry weight of seeds during germination [86,87].

The mobilization of seed storage proteins upon seed germination is a crucial process in seedling establishment. Plants often ensure early initiation of storage protein mobilization by depositing active proteases during seed maturation in the compartments where storage proteins are sequestered [88]. Breakdown of seed storage proteins can occur by a number of enzymes that possess proteolytic activity and catalyze an array of pathways. Degradation of globulins, i.e., 11S and 7S, occur by four papain-like cysteine proteases [89]. Protease C1, a serine endoprotease, mobilizes the accessible regions of 7S globulins and simultaneously preserves the core mainstream amino acids, including regions that are rich in glutamate and glutamine [90]. A 34 kDa Zn metalloproteinase is associated with the mobilization of 11S globulin in buckwheat (Fagopyrum esculentum), pumpkin and monocots [88]. Metalloproteases and aspartic proteases have been associated with the mobilization of prolamins in monocots during seed germination [91]. The degradation of seed storage proteins initiates the growth of seedlings because they serve as a source of nitrogen, help to sustain the growth of the shoot and provide amino acids and inorganic materials for biosynthesis [92,93].

## 7. Omics Approaches to Augment the Targeted Seed Storage Reserve Accumulation

**Figure 4:**
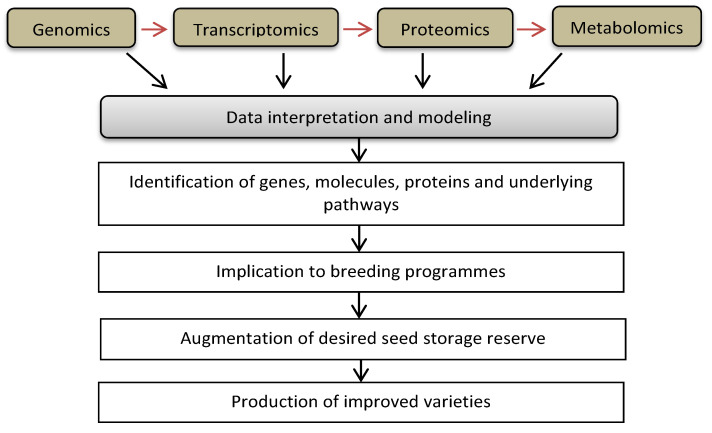
Omics approaches for the production of improved varieties in terms of the augmentation of seed storage reserves.

Understanding biochemical and molecular approaches integrated with omics, i.e., genomics, transcriptomics and proteomics, elucidates the key regulatory genes, molecules or proteins involved in the maturation of seeds, the accumulation of storage reserve and the development of seedling vigor. Targeting such molecules can be important for the augmentation of the desired storage reserve in relation to genetic improvement (Figure 4).

Various transcription factors or master regulators of seed composition can be identified by genomics and post-genomics research initiatives. Using microarray technology, ~21,000 genes involved in the grain filling process and metabolic pathways of carbohydrate and lipid synthesis have been identified in rice [94]. An Affymetrix 22K Barley1 gene chip analysis revealed 2020 genes involved in storage compound biosynthesis and cell development that are downregulated under heat stress [95]. In addition to transcriptomics, the posttranscriptional gene regulation of seed filling can be identified through different microRNAs (miRNAs). The crucial role of miRNAs as master regulators in regulating the seed filling process and controlling gene expression through the identification of their targets has been studied in diverse crops, such as maize, wheat and rice [96,97,98]. Specialized proteomics approaches could reveal important information regarding posttranslational switches that occur during seed maturation and reserve biosynthesis, and may yield more decisive results than traditional approaches. Quantitative trait loci could regulate the carbohydrate fillings of the seed, and genetic engineering can be used to inhibit or overexpress genes in the carbon flow pathway during starch, proteins or oil deposition in seeds, thus leading to the significant intensification of storage composites in numerous species [99,100,101,102]. Nuclear magnetic resonance imaging may be a useful tool for determining the overall distribution and changes in levels during seed development and reserve mobilization [103,104,105]. Storage reserves are markers of seed quality, and seedling vigor and metabolomics approaches can assess a broad range of metabolites responsible for their synthesis and accumulation, which may ultimately allow breeders to regulate a quantitative trait to produce valuable nutrients. Mutagenesis of starch branching enzymes facilitated by CRISPR/Cas9 generated rice with elevated amylose content [106]. The implications of such processes in breeding programs are the acceleration or augmentation of seed storage reserve accumulation in order to improve the nutritional quality of seeds.

## 8. Conclusions and Future Perspectives

During seed germination, storage reserves accumulate, which can ensure the robustness of the young seedling by aiding new plant establishment. As seeds accumulate these reserves, whose composition, type and amount depend upon the structure of the seeds and the type of species, the levels of storage reserves change under certain stages of seed development. Therefore, crop research at the biochemical, molecular and developmental levels is necessary to develop methods that can be used by biotechnologists and breeders to improve seed storage reserves in a practical manner. The signal transduction pathways for nutrient sensing that connect the changing levels of various intercompartmental seed storage reserves need to be elucidated. Interlinking seed storage accumulation with nitrogen metabolism can provide insights into the factors that control the segregation of photoassimilates into carbohydrates, lipids and proteins. The development of diverse, novel approaches, including biochemical, physiological and molecular approaches, will be helpful for augmenting seed storage reserves.

## Data Availability

Not applicable.
